# Parameterized Pseudo-Localization for Accurate and Efficient Moving Targets Imaging in Synthetic Aperture Radar

**DOI:** 10.3390/s17081714

**Published:** 2017-07-26

**Authors:** Xuepan Zhang, Lu Liu, Xuejing Zhang

**Affiliations:** 1 Qian Xuesen Laboratory of Space Technology, Beijing 100094, China; liulu@qxslab.cn; 2 School of Electronic Engineering, University of Electronic Science and Technology of China, Chengdu 611731, China; xjzhang7@163.com

**Keywords:** SAR, GMTI, moving targets imaging, Doppler delayed interferometry, Doppler rate estimation

## Abstract

Accurate and efficient moving target imaging is an important challenge for targets recognition in current synthetic aperture radar (SAR) combined with a ground moving target indication (GMTI) system. As the key but unknown parameter, the Doppler rates are estimated conventionally by searching any possible values for moving targets imaging. However, this conventional estimation method suffers from low accuracy or low efficiency due to the searching procedure. Focusing on these, we present a method to efficiently image the moving targets without the Doppler rate by Doppler delayed interferometry, and the imaged localization, which is parameterized pseudo-localization, is used to estimate the Doppler rate. In order to improve the estimation accuracy, an improved method based on the Newton method of approximation is proposed by exploiting the unused amplitude information. Compared with the conventional methods, the proposed improved method capable of high accuracy and low computation complexity simultaneously can meet the accurate and efficient requirements in the practical applications. Comparison simulations and real data processing results demonstrate the effectiveness of the proposed methods.

## 1. Introduction

Characterized by high resolution, cloud penetration, and remote sensing capabilities, synthetic aperture radar (SAR) has been studied intensively in both civil and military applications in recent years [1,2,3,4,5,6]. Combined with ground moving targets indication (GMTI) techniques, SAR-GMTI has been developed to be an effective and convenient way to realize moving target localization and recognition in the well-focused image domain [7,8,9,10,11,12]. In this case, the accurate focusing of the moving target becomes the most important prerequisite. It is well known that the focusing quality of the moving target is affected by the estimated Doppler rate [13]. A mismatch between the estimated Doppler rate and its real value not only causes severe blurring of the image but also degrades the target detection and localization performance [14]. Thus, accurate Doppler rate estimation is strongly desirable.

Composed of different Doppler rates due to different azimuth velocities [12], matched filter banks [15] are used in the range-Doppler algorithm (RDA) for moving target imaging. Image the moving target by any possible Doppler rate, and the one corresponding to the best focused result is selected as the Doppler rate of the moving target. Similar to matched filter banks, a time-frequency distribution that is expert in representing the modulate signal is often used to estimate the Doppler rate [14]. The fractional Fourier transform [16,17,18,19] and the Radon-Wigner distribution [19,20] can realize the Doppler rate estimation by searching all possible parameters. All of these conventional methods can be classified as being the search-based methods. However, the search-based methods are confronted with the following bottlenecks: (1) huge computation complexity due to searching all possible parameters; (2) when searching step size selection, there is a compromise when considering high estimation accuracy (due to small searching step size) and low computation complexity (introduced by large searching step size). These bottlenecks restrict the development of the search-based methods in practical applications, especially for the requirements of high real-time and high estimation accuracy. Summarily, the Doppler rate should be estimated as having high efficiency along with high accuracy.

Focusing on these, we present an efficient method to image the moving target without the Doppler rate, which is very different from the existing methods. Moreover, the imaged localization by the proposed method is parameterized pseudo-localization, which can be used to estimate the Doppler rate. In order to improve the estimation accuracy, we propose an improved method utilizing the Newton method of approximation by exploiting the unused amplitude information. Theoretical derivation and experiments results demonstrate the proposed methods with high accuracy and low computational complexity.

## 2. Signal Model for Moving Target in SAR System

After the range compression, the signal of the moving target can be expressed as

(1) $$\begin{array}{cc} S\left(\tau ,\eta \right) & =\sigma B_{r}\mathrm{sinc}(B_{r}\left(\tau -\frac{2}{c}\left(R_{0}+v_{r}\eta +\frac{{\left(x-\left(v-v_{a}\right)\eta \right)}^{2}}{2R_{0}}\right)\right))\times \\ & \exp\left[-j\frac{4\pi f_{c}}{c}\left(R_{0}+v_{r}\eta +\frac{{\left(x-\left(v-v_{a}\right)\eta \right)}^{2}}{2R_{0}}\right)\right] \end{array}$$

where σ is the radar cross section, $B_{r}$ and $f_{c}$ denote the bandwidth and the carrier frequency of the transmitted linear frequency modulated signal, respectively, τ and η represent the fast time and slow time. The radar platform keeps constant azimuth velocity v in the synthetic aperture time $T_{a}$, the moving target locates in the coordinate of $\left(x,R_{0}\right)$ at η=0, the radial velocity $v_{r}$ and the azimuth velocity $v_{a}$ of the moving target is also supposed to be constant in $T_{a}$.

Since the range walk due to the radial velocity of the moving target seriously affects the azimuth processing, we adopt the keystone transform [21,22] to compensate for the range walk. In order to realize the keystone transform, the range compression result (1) is transformed into the range frequency domain by the fast Fourier transform (FFT) in the range

(2) $$S\left(f_{r},\eta \right)=\sigma \exp\left[-j\frac{4\pi \left(f_{c}+f_{r}\right)}{c}\left(R_{0}+v_{r}\eta +\frac{{\left(x-\left(v-v_{a}\right)\eta \right)}^{2}}{2R_{0}}\right)\right]$$

The following relationship

(3) $$\left(f_{c}+f_{r}\right)\eta =f_{c}\eta^{\prime }$$

is substituted into (2), and then we can obtain

(4) $$\begin{array}{cc} S\left(f_{r},\eta^{\prime }\right) & =\sigma \exp\left(-j\frac{4\pi f_{c}}{c}\left(v_{r}-\frac{x\left(v-v_{a}\right)}{R_{0}}\right)\eta^{\prime }\right)\times \\ & \exp\left(-j\frac{4\pi \left(f_{c}+f_{r}\right)}{c}\left(R_{0}+\frac{x^{2}}{2R_{0}}\right)\right)\exp\left(-j\frac{2\pi }{c}\frac{{\left(v-v_{a}\right)}^{2}}{R_{0}}\frac{f_{c}^{2}{\eta^{\prime }}^{2}}{f_{c}+f_{r}}\right) \end{array}$$

Using the following approximation [23]

(5) $$\frac{1}{f_{c}+f_{r}}\approx \frac{1}{f_{c}}-\frac{f_{r}}{f_{c}^{2}},\;f_{r}<<f_{c}$$

(4) can be rewritten as

(6) $$\begin{array}{cc} S\left(f_{r},\eta^{\prime }\right) & =\sigma \exp\left(-j\frac{4\pi f_{c}}{c}\left(v_{r}-\frac{x\left(v-v_{a}\right)}{R_{0}}\right)\eta^{\prime }\right)\exp\left(-j\frac{4\pi \left(f_{c}+f_{r}\right)}{c}\left(R_{0}+\frac{x^{2}}{2R_{0}}\right)\right)\times \\ & \exp\left(-j\frac{2\pi }{c}\frac{{\left(v-v_{a}\right)}^{2}f_{c}{\eta^{\prime }}^{2}}{R_{0}}\right)\exp\left(j\frac{2\pi }{c}\frac{{\left(v-v_{a}\right)}^{2}f_{r}{\eta^{\prime }}^{2}}{R_{0}}\right) \end{array}$$

In order to compensate for the range curve effect, we adopt the following procedure

(7) $$\begin{array}{cc} S^{\prime }\left(f_{r},\eta^{\prime }\right) & =S\left(f_{r},\eta^{\prime }\right)\cdot \exp\left(-j\frac{2\pi }{c}\frac{v^{2}f_{r}{\eta^{\prime }}^{2}}{R_{0}}\right)\\ & =\sigma \exp\left(-j\frac{4\pi f_{c}}{c}\left(v_{r}-\frac{x\left(v-v_{a}\right)}{R_{0}}\right)\eta^{\prime }\right)\exp\left(-j\frac{2\pi }{c}\frac{{\left(v-v_{a}\right)}^{2}f_{c}{\eta^{\prime }}^{2}}{R_{0}}\right)\times \\ & \exp\left(-j\frac{4\pi \left(f_{c}+f_{r}\right)}{c}\left(R_{0}+\frac{x^{2}}{2R_{0}}\right)\right)\exp\left(j\frac{2\pi }{c}\frac{\left({\left(v-v_{a}\right)}^{2}-v^{2}\right)f_{r}{\eta^{\prime }}^{2}}{R_{0}}\right) \end{array}$$

Applying the inverse FFT (IFFT) in range, the range compression result after range walk and curve correction can be written as

(8) $$\begin{array}{cc} S\left(\tau ,\eta^{\prime }\right) & =\sigma B_{r}\mathrm{sinc}\left(B_{r}\left(\tau -\frac{2R_{0}}{c}-\frac{2x^{2}}{cR_{0}}+\frac{\left({\left(v-v_{a}\right)}^{2}-v^{2}\right){\eta^{\prime }}^{2}}{cR_{0}}\right)\right)\exp\left(-j\frac{4\pi f_{c}}{c}\left(R_{0}+\frac{x^{2}}{2R_{0}}\right)\right)\times \\ & \exp\left(-j\frac{4\pi f_{c}}{c}\left(v_{r}-\frac{x\left(v-v_{a}\right)}{R_{0}}\right)\eta^{\prime }\right)\exp\left(-j\frac{2\pi f_{c}}{c}\frac{{\left(v-v_{a}\right)}^{2}{\eta^{\prime }}^{2}}{R_{0}}\right)\\ & \approx \sigma B_{r}\mathrm{sinc}\left(B_{r}\left(\tau -\frac{2R_{0}}{c}\right)\right)\exp\left(-j\frac{4\pi f_{c}}{c}\left(R_{0}+\frac{x^{2}}{2R_{0}}\right)\right)\times \\ & \exp\left(-j\frac{4\pi f_{c}}{c}\left(v_{r}-\frac{x\left(v-v_{a}\right)}{R_{0}}\right)\eta^{\prime }\right)\exp\left(-j\frac{2\pi f_{c}}{c}\frac{{\left(v-v_{a}\right)}^{2}{\eta^{\prime }}^{2}}{R_{0}}\right) \end{array}$$

where the approximation holds because

$$\frac{2x^{2}}{cR_{0}}-\frac{\left({\left(v-v_{a}\right)}^{2}-v^{2}\right){\eta^{\prime }}^{2}}{cR_{0}}<<\frac{2R_{0}}{c}$$

It can be seen from (8) that the Doppler rate of the moving target

(9) $$\gamma_{a}=-\frac{2f_{c}}{c}\frac{{\left(v-v_{a}\right)}^{2}}{R_{0}}$$

is unknown because of its unknown $v_{a}$. Since the Doppler rate of the moving target is different from that of the stationary targets (or scenery), the moving target would be defocused when imaging the scenery with the Doppler rate of stationary targets. It is known that the Doppler rate plays an important role in not only moving target imaging but also the azimuth velocity estimation, both of which can be used for moving target recognition. Moreover, the Doppler rate estimation accuracy determines the performance of moving target imaging and velocity estimation. Thus, the Doppler rate should be estimated as accurately as possible.

Conventionally, any possible Doppler rates are used to realize the azimuth compression for moving target imaging, and the one corresponding to the best focused result can be thought as the most accurate estimated Doppler rate. And then the moving target imaging and its azimuth velocity can be obtained. This is the main idea of the conventional Doppler rate estimation method. However, since the matched Doppler rate is searched from all of possible chirp rates, the computation complexity is huge, and the estimation accuracy is seriously affected by the searching step size.

## 3. The Proposed Parameterized Pseudo-Localization for Efficient Doppler rate Estimation

Focusing on these, we have proposed a new Doppler rate estimation method by using the localization information. Different from the existing searching based method, the proposed method can achieve efficient estimation. Moreover, these existing estimation methods achieve Doppler rate estimation after moving target imaging, while the proposed method realizes moving target imaging inventively without Doppler rate, and the Doppler rate can be estimated from the localization of the imaged moving target.

Without Doppler rate, the moving target cannot be focused well by the range-Doppler algorithm (RDA). Being cognizant of this, we utilize the Doppler delay interferometry (DDI) to eliminate the Doppler rate term, which is derived in detail as follows.

Firstly, the range compression result in (8) is transformed by FFT in azimuth into the Doppler domain as

(10) $$\begin{array}{cc} S\left(\tau ,f_{a}\right) & =\sigma B_{r}\mathrm{sinc}\left(B_{r}\left(\tau -\frac{2R_{0}}{c}\right)\right)\exp\left(-j\frac{4\pi f_{c}}{c}\left(R_{0}+\frac{x^{2}}{2R_{0}}\right)\right)\exp\left(\frac{j\pi f_{a}^{2}cR_{0}}{2f_{c}{\left(v-v_{a}\right)}^{2}}\right)\times \\ & \exp\left(j\frac{2\pi f_{c}}{c}{\left(v_{r}-\frac{x\left(v-v_{a}\right)}{R_{0}}\right)}^{2}\frac{R_{0}}{{\left(v-v_{a}\right)}^{2}}\right)\cdot \exp\left(\frac{j2\pi f_{a}R_{0}}{{\left(v-v_{a}\right)}^{2}}\left(v_{r}-\frac{x\left(v-v_{a}\right)}{R_{0}}\right)\right) \end{array}$$

Its Doppler delayed result can be written as

(11) $$\begin{array}{cc} S\left(\tau ,f_{a}+\Delta f_{a}\right) & =\sigma B_{r}\mathrm{sinc}\left(B_{r}\left(\tau -\frac{2R_{0}}{c}\right)\right)\exp\left(-j\frac{4\pi f_{c}}{c}\left(R_{0}+\frac{x^{2}}{2R_{0}}\right)\right)\exp\left(\frac{j\pi {\left(f_{a}+\Delta f_{a}\right)}^{2}cR_{0}}{2f_{c}{\left(v-v_{a}\right)}^{2}}\right)\times \\ & \exp\left(j\frac{2\pi f_{c}}{c}{\left(v_{r}-\frac{x\left(v-v_{a}\right)}{R_{0}}\right)}^{2}\frac{R_{0}}{{\left(v-v_{a}\right)}^{2}}\right)\exp\left(\frac{j2\pi \left(f_{a}+\Delta f_{a}\right)R_{0}}{{\left(v-v_{a}\right)}^{2}}\left(v_{r}-\frac{x\left(v-v_{a}\right)}{R_{0}}\right)\right) \end{array}$$

where $\Delta f_{a}$ denotes the delayed interval in the Doppler domain. Interferometry is usually used to obtain the different information between two signals, and the interferometry SAR (InSAR) [24] is the well-known application by interferometry between two channels: for example, along-track interferometry (ATI) [25,26,27,28] used for moving targets detection or motion estimation, and cross-track interferometry used for digital elevation model [29,30,31]. Here, interferometry is done between (10) and (11) to eliminate the same Doppler rate terms, and then the DDI result can be derived as

(12) $$\begin{array}{cc} \Delta S\left(\tau ,f_{a}\right) & =S^{*}\left(\tau ,f_{a}\right)\cdot S\left(\tau ,f_{a}+\Delta f_{a}\right)\\ & =\sigma^{2}B_{r}^{2}{\mathrm{sinc}}^{2}\left(B_{r}\left(\tau -\frac{2R_{0}}{c}\right)\right)\exp\left(\frac{j\pi \left(2f_{a}\Delta f_{a}+\Delta f_{a}^{2}\right)cR_{0}}{2f_{c}{\left(v-v_{a}\right)}^{2}}\right)\times \\ & \exp\left(j2\pi \Delta f_{a}\left(v_{r}-\frac{x\left(v-v_{a}\right)}{R_{0}}\right)\frac{R_{0}}{{\left(v-v_{a}\right)}^{2}}\right) \end{array}$$

where $S^{*}\left(\tau ,f_{a}\right)$ stands for the conjugate of $S\left(\tau ,f_{a}\right)$. It can be seen from (12) that the Doppler quadratic term is eliminated by the DDI, which can be thought as the concept of self-match. Since the Doppler rate term is removed, the moving target imaging result can be obtained by the IFFT in azimuth as

(13) $$\begin{array}{cc} \Delta S\left(\tau ,\eta \right) & =\sigma^{2}B_{r}^{2}B_{a}{\mathrm{sinc}}^{2}\left(B_{r}\left(\tau -\frac{2R_{0}}{c}\right)\right)\mathrm{sinc}\left(B_{a}\left(\eta +\frac{\Delta f_{a}cR_{0}}{2f_{c}{\left(v-v_{a}\right)}^{2}}\right)\right)\times \\ & \exp\left(j2\pi \Delta f_{a}\left(v_{r}-\frac{x\left(v-v_{a}\right)}{R_{0}}\right)\frac{R_{0}}{{\left(v-v_{a}\right)}^{2}}\right)\exp\left(\frac{j\pi \Delta f_{a}^{2}cR_{0}}{2f_{c}{\left(v-v_{a}\right)}^{2}}\right)\\ & =\sigma^{2}B_{r}^{2}B_{a}{\mathrm{sinc}}^{2}\left(B_{r}\left(\tau -\frac{2R_{0}}{c}\right)\right)\mathrm{sinc}\left(B_{a}\left(\eta -\frac{\Delta f_{a}}{\gamma_{a}}\right)\right)\times \\ & \exp\left(j2\pi \Delta f_{a}\left(v_{r}-\frac{x\left(v-v_{a}\right)}{R_{0}}\right)\frac{R_{0}}{{\left(v-v_{a}\right)}^{2}}\right)\exp\left(\frac{j\pi \Delta f_{a}^{2}cR_{0}}{2f_{c}{\left(v-v_{a}\right)}^{2}}\right) \end{array}$$

By focusing only on the amplitude term related with the azimuth localization, we can rewrite the moving target imaging result into

(14) $$\left|\Delta S\left(\tau ,\eta \right)\right|=K\left(\tau \right)\cdot \mathrm{sinc}\left(B_{a}\left(\eta -\frac{\Delta f_{a}}{\gamma_{a}}\right)\right)$$

where K(τ) denotes the other amplitude terms except the azimuth sinc function. It can be seen from (14) that the original azimuth localization x is not contained in (14), that is, it is pseudo-localization. This is because the DDI eliminate the Doppler centroid which including x. Optimistically, coins have two sides. The pseudo-localization is determined by the unknown Doppler rate of the moving target $\gamma_{a}$, so the Doppler rate of the moving target can be estimated by the pseudo-localization of the moving target via

(15) $${\hat{\gamma }}_{a}=-\Delta f_{a}/\eta_{0}$$

where $\eta_{0}$ is the measured pseudo-localization of the moving target, which corresponds to the max amplitude in the imaging result of (13).

Visually, the estimation accuracy of the Doppler rate is intimately affected by the measurement accuracy of the pseudo-localization. However, since the azimuth resolution is not very high due to the limited Doppler bandwidth, the measured pseudo-localization of the moving target is not accurate, namely,

(16) $$\eta_{0}=round\left(\eta_{m}\cdot PRF\right)/PRF$$

where $\eta_{m}$ denotes the theoretical localization, the function of $round\left(\eta_{m}\cdot PRF\right)$ rounds the elements of $\eta_{m}\cdot PRF$ to the nearest integer. At that point the Doppler rate can be obtained.

In this section, the proposed method is presented to image the moving targets without the Doppler rate, and its parameterized pseudo-localization is modeled to estimate the Doppler rate efficiently. However, due to the round operation, the localization $\eta_{0}$ is measured with error, and then the Doppler rate is estimated in low accuracy. In the next section, we will propose an improved method to realize much more accurate localization measurement, and then the Doppler rate can be estimated with much higher accuracy.

## 4. The Improved Accurate Estimation Method

In order to measure the localization as accurately as possible, we utilize Newton method of approximation by using the amplitude information of the moving target imaging result.

By expanding the sinc function, we can rewrite the moving target imaging result in (14) as

(17) $$\left|\Delta S\left(\tau ,\eta \right)\right|=K\left(\tau \right)\cdot \frac{\sin\left(\pi B_{a}\left(\eta -\eta_{m}\right)\right)}{\pi B_{a}\left(\eta -\eta_{m}\right)}$$

Considering the discrete representation, we can express the slow time η by

(18) $$\eta =\frac{n}{PRF}$$

Since the amplitude term K is constant but unknown, we adopt two amplitudes to eliminate the constant term K. And the amplitudes can be written by

(19) $$\left|\Delta S\left(\tau ,\frac{n}{PRF}\right)\right|=K\left(\tau \right)\cdot \frac{\sin\left(\pi B_{a}\left(\frac{n}{PRF}-\eta_{m}\right)\right)}{\pi B_{a}\left(\frac{n}{PRF}-\eta_{m}\right)}=a_{n}$$

(20) $$\left|\Delta S\left(\tau ,\frac{n+1}{PRF}\right)\right|=K\left(\tau \right)\cdot \frac{\sin\left(\pi B_{a}\left(\frac{n+1}{PRF}-\eta_{m}\right)\right)}{\pi B_{a}\left(\frac{n+1}{PRF}-\eta_{m}\right)}=a_{n+1}$$

where $a_{n}$ and $a_{n+1}$ denote the amplitude corresponding to azimuth localization $\frac{n}{PRF}$ and $\frac{n+1}{PRF}$, respectively. Division between (19) and (20) is done to eliminate K(τ) through

(21) $$\frac{\sin\left(\pi B_{a}\left(\frac{n}{PRF}-\eta_{m}\right)\right)}{\sin\left(\pi B_{a}\left(\frac{n+1}{PRF}-\eta_{m}\right)\right)}\frac{\pi B_{a}\left(\frac{n+1}{PRF}-\eta_{m}\right)}{\pi B_{a}\left(\frac{n}{PRF}-\eta_{m}\right)}=\frac{a_{n}}{a_{n+1}}$$

In order to use the Newton method, the division result of (21) can be rewritten as

(22) $$f\left(\eta_{m}\right)=a_{n+1}\pi B_{a}\left(\frac{n+1}{PRF}-\eta_{m}\right)\sin\left(\pi B_{a}\left(\frac{n}{PRF}-\eta_{m}\right)\right)-a_{n}\pi B_{a}\left(\frac{n}{PRF}-\eta_{m}\right)\sin\left(\pi B_{a}\left(\frac{n+1}{PRF}-\eta_{m}\right)\right)$$

Using the Newton method to solve (22), we can obtain the accurate localization $\eta_{m}$ by the following iteration

(23) $${\hat{\eta }}_{m}^{k}={\hat{\eta }}_{m}^{k-1}-\frac{f\left(\eta_{m}\right)}{f^{\prime }\left(\eta_{m}\right)}$$

where $f^{\prime }\left(\eta_{m}\right)$ denotes the differentiate of $f\left(\eta_{m}\right)$, ${\hat{\eta }}_{m}^{k}$ represents the kth iterated localization, and the original value of ${\hat{\eta }}_{m}^{0}$ can be set as $\frac{n}{PRF}$. We can set the terminated condition according to different requirements. For example, the iterated results vary slightly as $\left|{\hat{\eta }}_{m}^{k}-{\hat{\eta }}_{m}^{k-1}\right|<10^{-6}$ or the iterated times is large enough, such as k>100. After the iteration is terminated, we can obtain the accurate localization ${\hat{\eta }}_{m}^{k}$, and then the Doppler rate of the moving target can be calculated by

(24) $${\hat{\gamma }}_{a}=\pi B_{a}\frac{\Delta f_{a}}{{\hat{\eta }}_{m}^{k}}$$

Since the Newton method can be realized efficiently and accurately, the Doppler rate of the moving target can be estimated by the proposed method with high accuracy and low computation complexity. After obtaining the Doppler rate of the moving target, we can image the moving target by RDA, and then the real localization can be obtained.

Summarily, by exploiting the amplitude information, the proposed improved method simultaneously possesses the advantages of high accuracy and low computational complexity.

## 5. Experiment Results and Analysis

In this section, the experiments results are presented to demonstrate the effectiveness of the proposed methods. The system parameters are shown in Table 1.

### 5.1. Moving Target Imaging Comparison between the RDA and the Presented DDI

**Figure 1:**
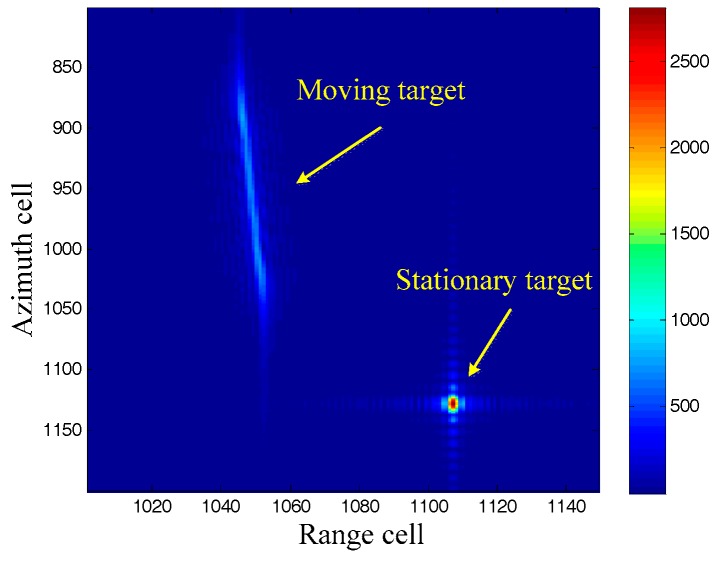
Imaging results by range-Doppler algorithm (RDA).

**Figure 2:**
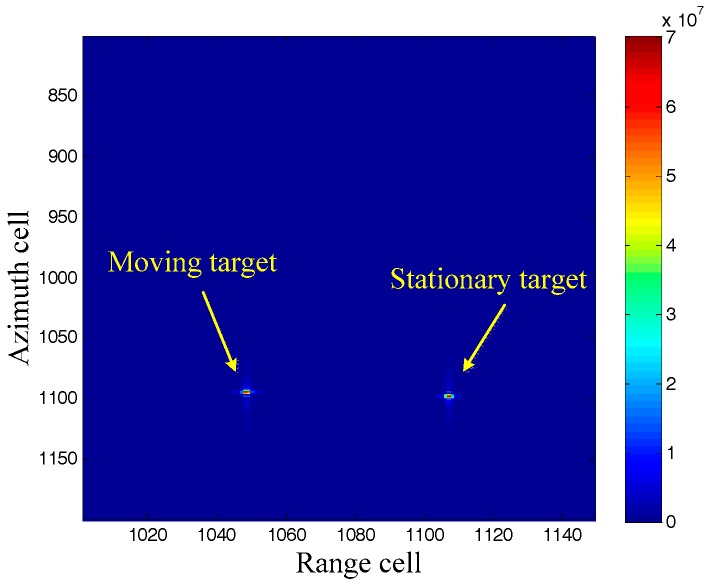
Imaging results by Doppler delay interferometry (DDI).

In order to present the advantages of the DDI, we compare the moving targets imaging between the RDA and the DDI in this subsection. A moving target and a stationary target, with the same azimuth localization x, are imaged by both the RDA and the DDI. The RDA is realized by using the Doppler rate of the stationary target, which can be easily obtained by the system parameters. The imaging results are shown in Figure 1 and Figure 2.

**Figure 3:**
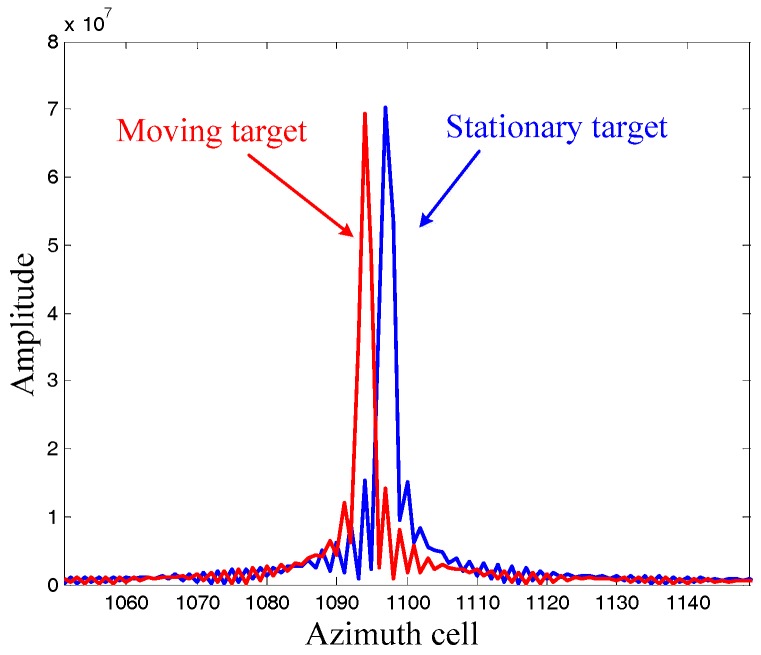
Azimuth localization comparison.

It can be seen from Figure 1 that the stationary target is well focused, while the moving target is badly defocused because of its different Doppler rate. Moreover, the imaging moving target is displaced from its original azimuth localization x due to its radial velocity. We will now pay attention to the imaging results by the DDI in Figure 2. Firstly, both of the moving target and the stationary target are focused well, demonstrating that the DDI method can realize focused imaging results with an unknown Doppler rate. Secondly, the amplitudes of the targets imaged by the DDI are much higher than that by the RDA. This is because the DDI enhances the amplitude by the interferometry according to (12), which is very useful for improving the signal to noise ratio (SNR) in practical applications. Thirdly, the azimuth localization of the moving target imaged by the DDI is also displaced from its original localization. The azimuth localization comparison between the moving target and the stationary target, with the same original azimuth localization, is shown as Figure 3.

It can be seen that the azimuth localization of the moving target is different from that of the stationary target, that is, the azimuth localization imaged by DDI is pseudo-localization. This is because the Doppler rates of for them are different from each other, which has been aforementioned in the theoretical derivation. Moreover, the foundation of the Doppler rate estimation by this pseudo-localization has also been well founded.

### 5.2. Doppler Rate Estimation Performance Comparison

**Figure 4:**
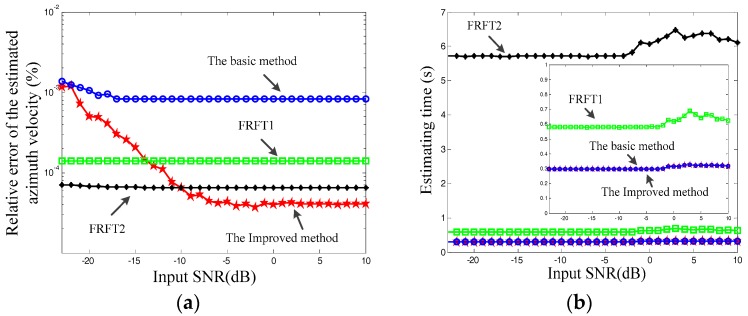
Doppler rate estimation comparison results. (**a**) estimation accuracy comparison; (**b**) computational complexity comparison.

In this subsection, the Doppler rate estimation accuracy versus to the SNR of the echo is simulated. The proposed basic method, the improved method and the conventional fractional Fourier transform (FRFT) are compared to estimate the Doppler rate, with the comparison results shown in Figure 4. It is known that the FRFT can be used to estimate the Doppler rate by searching any possible values, but the estimation accuracy and computational complexity should be a trade-off due to the searching step size. In order to show these properties of the conventional FRFT, we adopt two FRFTs with different searching step sizes: method 1 (FRFT1) with a searching step size of the FRFT order as 0.5, and; method 2 (FRFT2) with a step size of 0.05.

It can be seen from Figure 4a,b that the improved method possesses much higher estimation accuracy than the basic method, but with almost the same computational complexity. This is because the basic method estimates the Doppler rate by using integer localization, while the improved method can utilize much more accurate localization obtained by the Newton method without the complexity burden increasing. The following trade-off properties of the conventional FRFT methods can be seen from Figure 4: a large step size brings low complexity with low accuracy, while small step size brings high accuracy with high complexity. Compared with the conventional FRFT methods, the basic method possesses much higher efficiency with much lower accuracy, while the improved method presents much better in both terms of estimation and computational complexity, which shows the advantages of the proposed improved method.

**Figure 5:**
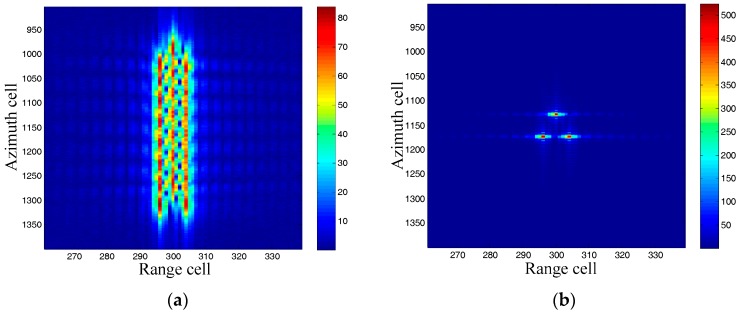
Moving targets imaging results. (**a**) by RDA with Doppler rate of stationary parameters, and (**b**) re-imaging results by the estimated Doppler rate.

After Doppler rate estimation, we focus on moving targets imaging results by the estimated Doppler rate. A moving target with three scatters is simulated to demonstrate the effectiveness of the proposed method in moving target imaging and recognition, and its azimuth velocity is set as 20 m/s with radial velocity of zero. The RDA is done with the Doppler rate obtained by the stationary parameters, and the proposed improved method is used to estimate the Doppler rate first, then the RDA is used to re-image the moving target with the estimated Doppler rate, with the results shown in Figure 5.

It can be seen from Figure 5a that the moving target imaging results by the RDA with Doppler rate of stationary parameters is unfocused with low energy in its spread along azimuth cells. And the re-imaging results are well focused due to the well-estimated Doppler rate by the proposed improved method, which can be used for the targets recognition.

### 5.3. Real Data Process

**Figure 6:**
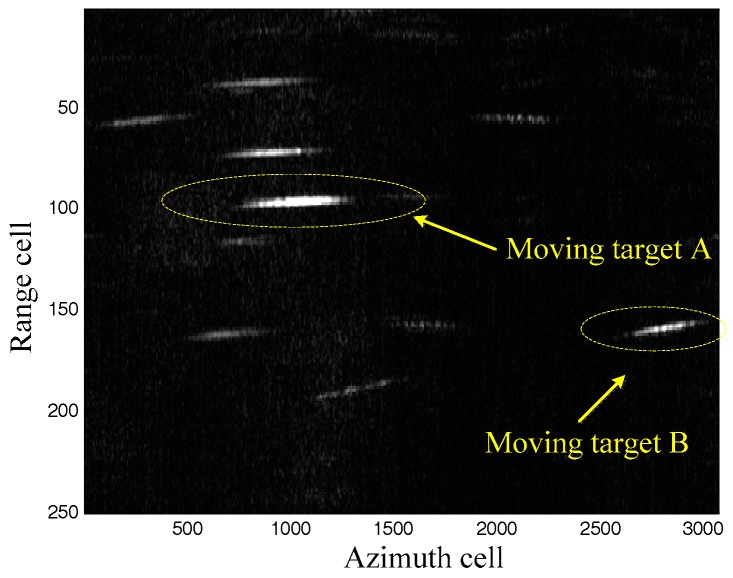
Clutter suppression results.

**Figure 7:**
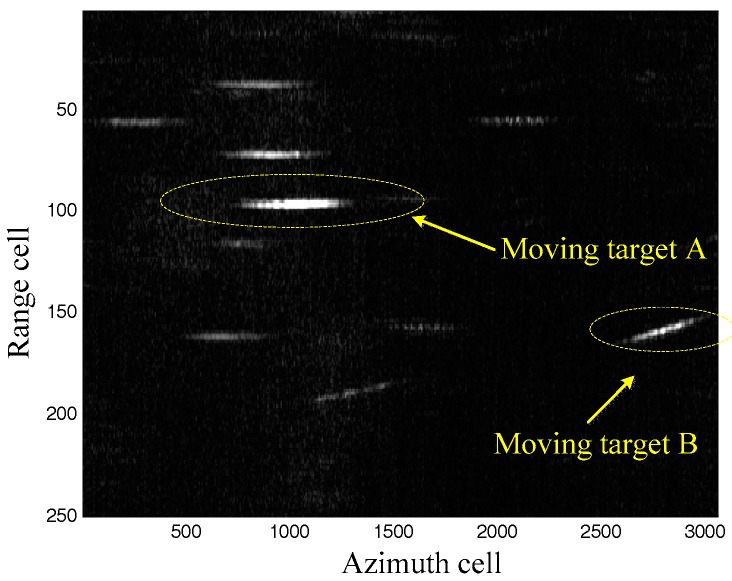
Keystone transform results.

Real data is processed to further demonstrate the effectiveness of the proposed method. The system parameters are listed in Table 1. The clutter suppression result by the extended factored approach [32,33,34] in the range-Doppler domain is shown as Figure 6. The moving targets are easy to detect. Moreover, the range walk due to the radial velocity of the moving targets appears in Figure 6, which should be corrected to guarantee the moving targets imaging. As aforementioned, the keystone transform is used to realize the range walk correction, with the results shown in Figure 7. It can be seen that the range walk is effectively corrected for moving target A, while the same consequence does not occur for moving target B. This is because the used keystone transform is valid for the targets with the unambiguous radial velocities but invalid for those with ambiguous radial velocities (for example, moving target B).

**Figure 8:**
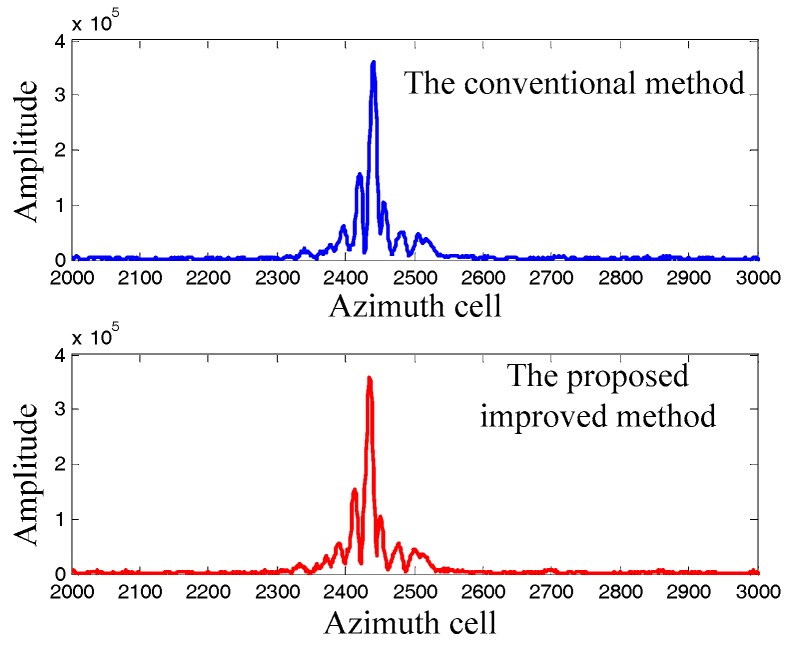
Moving target imaging results comparison.

Let us take the moving target A as an example. The proposed improved method and the conventional method are used to estimate the Doppler rate, with the estimation results of ${\hat{\gamma }}_{a}^{p}=-81.38\;\mathrm{Hz/}s^{2}$ and ${\hat{\gamma }}_{a}^{c}=-81.61\;\mathrm{Hz/}s^{2}$, respectively. The Doppler rate estimation results are used to reimage the moving target A by the RDA, and the imaging results are compared in Figure 8. It can be seen that the imaging results are similar to each other, which concludes that the proposed method is effective in the real scenarios.

## 6. Conclusions

In this paper, we have proposed a parametrized pseudo-localization based Doppler rate estimation to realize moving targets imaging in high accuracy and low computational complexity. The proposed basic method can image the moving targets without the Doppler rate, and the imaged localization derived as the parameterized pseudo-localization can be used to estimate the Doppler rate in low computational complexity. The improved method is proposed to improve the estimation accuracy. The amplitude information of the imaged results is exploited, and the Newton method of approximation is used to obtain the Doppler rate with much higher accuracy. The advantages of the proposed methods are validated by the comparison experiments results. Compared with the conventional estimation methods, the proposed methods with high accuracy and low computation load can meet the accuracy and efficiency requirements of practical applications.

## Figures and Tables

**Table 1:** System parameters.

System Parameters	Values
Carrier frequency	8.85 GHz
Number of the channels	3
Channels space	0.56 m
Bandwidth of the transmitted signal	40 MHz
Sampling frequency	60 MHz
Velocity of the platform	120 m/s
Pulse repetition frequency	1000 Hz
Nearest slant range	9000 m
